# Internet of Medical Things Enabled Multimodal Framework: Deep Machine Learning for Chronic Cardiac Disease Prediction in Healthcare 5.0

**DOI:** 10.1049/htl2.70063

**Published:** 2026-02-10

**Authors:** Rabia Javed, Tahir Abbas, Ali Sayyed, Sagheer Abbas, Asghar Ali Shah, Khan Muhammad Adnan

**Affiliations:** ^1^ Department of Computer Science TIMES University Multan Pakistan; ^2^ Department of Communication and Cyber Security Bahauddin Zakariya University Multan Pakistan; ^3^ National University of Computer and Emerging Sciences, Hayatabad Peshawar Pakistan; ^4^ Prince Mohammad Bin Fahad University, Alkhobar Dhahran Saudi Arabia; ^5^ Department of Computer Science Kateb University Kabul Afghanistan; ^6^ Department of Software Faculty of Artificial Intelligence and Software Gachon University Seongnam‐si Republic of Korea

**Keywords:** conceptualization, formal analysis, investigation, methodology, software, validation, visualization, writing, original draft

## Abstract

Accurate and early detection of chronic heart disease is vital, as it remains one of the leading global causes of mortality. Despite advancements in Smart Healthcare 5.0 and modern information technologies, reliable diagnosis of cardiovascular conditions remains a significant challenge. The Internet of Medical Things (IoMT) enables seamless data exchange between medical devices, supporting more precise and timely management of cardiac diseases. This study employs convolutional neural networks (CNNs) on electrocardiogram (ECG) image datasets to classify multiple heart conditions. The datasets include ECG scans labelled as Abnormal Heartbeat (ANHB), Myocardial Infarction (MI), History of Myocardial Infarction (HOMI), Atrioventricular Heart Block (AHB), COVID‐19, Hypertrophic Cardiomyopathy (HMI), and Normal. A multimodal model integrating images of varying resolutions from two independent datasets was developed to improve classification performance. The proposed CNN model, trained and validated on preprocessed ECG images, achieved 97.18% training accuracy and 94.34% validation accuracy. By combining ECG data from diverse sources, the model enhances the identification of cardiac irregularities and provides a comprehensive diagnostic approach. This method demonstrates potential to support early detection, improve individualised treatment planning, and ultimately strengthen patient outcomes in managing chronic heart disease.

## Graphical abstract

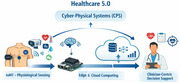

## 1 Introduction

The rise in chronic cardiac diseases, including heart failure, arrhythmias, and coronary artery disease, presents a significant global health challenge. This situation underscores the need for novel strategies aimed at early identification and treatment. The convergence of the Internet of Medical Things (IoMT) and Healthcare 5.0, along with cutting‐edge technologies like deep learning, offers enhanced diagnostic and predictive capabilities that could transform the medical industry. This study focuses on applying deep learning algorithms within an IoMT‐enabled Healthcare 5.0 framework for multimodal electrocardiogram (ECG) image classification aimed at diagnosing chronic heart diseases.

The healthcare sector and academic institutions are closely monitoring advancements in smart medical sensors, equipment, cloud computing, and healthcare technologies. Medical image processing, particularly, remains one of the most dynamic research areas in healthcare. The Internet of Things (IoT) has gained recognition for its role in this domain, giving rise to the Internet of Medical Things (IoMT), a network of intelligent medical devices connected to the internet. These devices, including glucose meters, heart monitors, and fitness trackers, gather essential health data, such as blood sugar levels and pulse rate. This data is automatically transmitted to hospitals or doctors, reducing the need for in‐person visits. Traditional medical devices like Computed Tomography (CT) and Magnetic Resonance Imaging (MRI) machines, as well as hospital resources such as medication bottles and medical records, are also integrated into the IoMT ecosystem [1]. When integrated with the latest technologies like AI, deep learning and machine learning, can better protect public health. It improves patient comfort, offers cost‐effective medical solutions, expedites hospital treatments, and provides more personalised healthcare [2].

IoMT systems allow for the remote monitoring of patients with chronic diseases, enabling timely diagnostics in emergencies, which can be life‐saving [3]. The field of IoMT has also advanced rapidly, supporting the collection, processing, and analysis of medical data generated by numerous IoT devices [4]. This network of interconnected medical devices uses the cloud to transmit data [5]. As these devices are networked and integrated, they can exchange and gather data using various standards and technologies. Furthermore, recent developments in IoMT‐based healthcare systems have shown how well wearable sensors, edge‐enabled AI, and predictive analytics may be integrated for real‐time patient monitoring and decision assistance. In order to facilitate continuous remote monitoring, lessen hospital effort, and assist early clinical intervention, recent research has documented IoMT frameworks that integrate physiological sensors (such as heart rate, temperature, and blood oxygen saturation) with AI‐driven analytics and edge devices [6]. Consequently, IoMT is seen as a solution to the shortage of medical resources and has helped reduce hospital visits [7].

**FIGURE 1:**
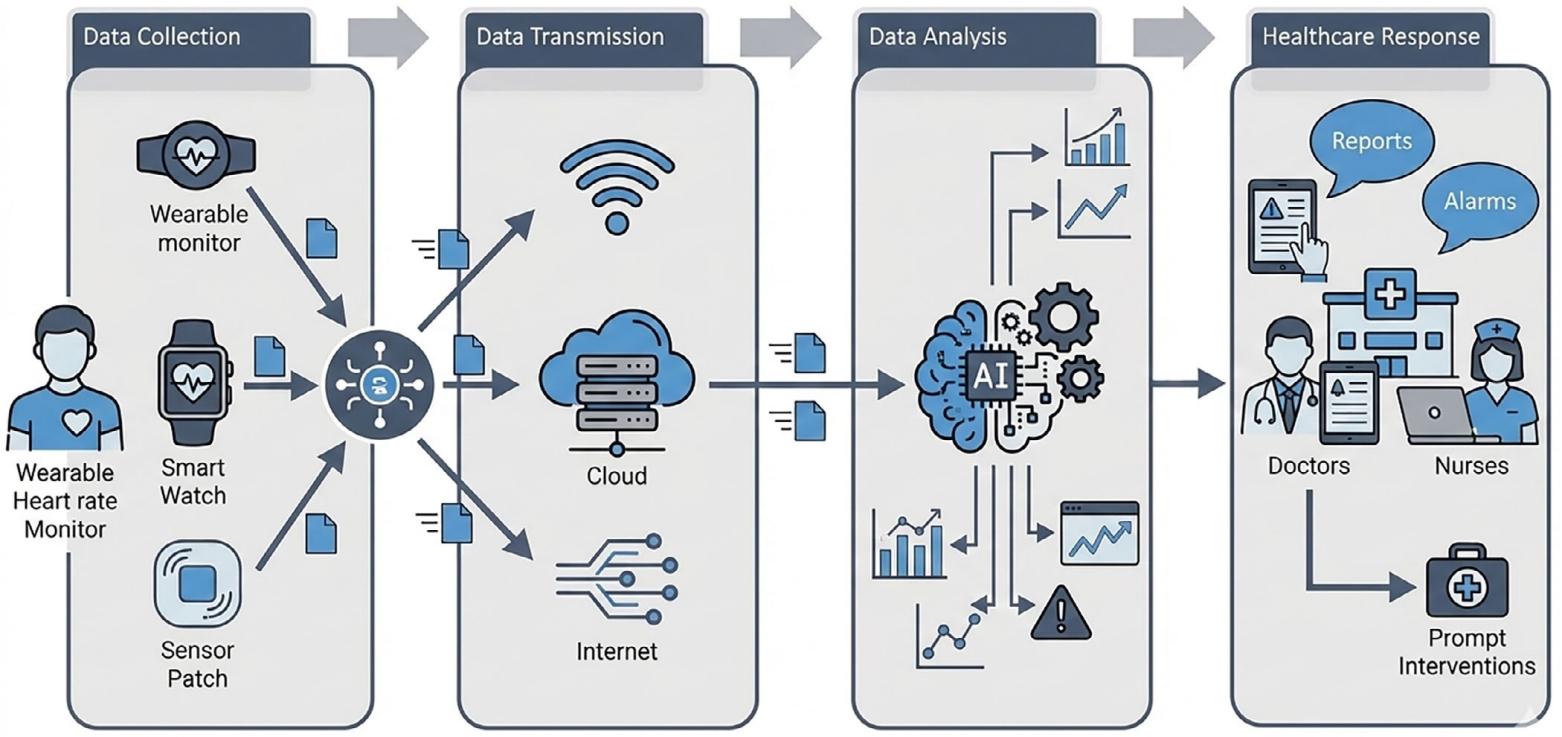
Workflow of Internet of Medical Things (IoMT).

Figure 1 represents the working of IoMT. The process starts with data collection from IoMT devices like fitness trackers, ECG monitors, glucose monitors, pulse oximeters, MRIs, and many other sensors and medical devices. This collected data is gathered and transmitted to the cloud or servers; and advanced AI model performs analysis, and lastly, this evaluated data is sent to healthcare professionals for making prompt interventions and making recommendations.

To analyse medical images, researchers have employed artificial intelligence, machine learning, and deep learning approaches. These advanced methods assist physicians in identifying and diagnosing diseases at an earlier stage, providing precise, reliable, efficient, and timely care that can help reduce mortality rates. Globally, more than 64 million people are affected by heart disease, and both its prevalence and incidence are steadily rising. Heart disease patients often experience a reduced quality of life and face premature death [8]. Cardiac disease has already imposed a financial burden of over $100 billion on the global economy. In response, international guidelines strongly recommend measures to prevent the progression of cardiac disease. Early diagnosis of these conditions is essential to implementing preventive actions and mitigating serious incidents [9].

Effective management of cardiovascular health relies on prevention, which includes reducing risk factors and emphasising early detection [10]. Cardiovascular disease (CVD) has become a significant public health concern with substantial socioeconomic costs for governments, patients’ families, and society as a whole. Risk stratification algorithms can identify patients at high risk of CVD, allowing for targeted interventions, such as dietary modifications, that help lower this risk and support CVD prevention efforts [11]. Chronic diseases, which include diabetes, heart disease, and chronic respiratory disorders, are major causes of mortality. To lessen the effects of these disorders, early detection and treatment are essential. However, conventional diagnostic techniques frequently fall short of the accuracy and promptness required for successful intervention, which makes the use of cutting‐edge technologies necessary [12].

Cardiovascular imaging is one of the fields showing significant interest in artificial intelligence (AI). Among the various diagnostic imaging modalities, machine learning (ML) is demonstrating great potential. Due to current limitations in processing power, traditional statistical methods are being replaced by machine learning, which opens up new possibilities and reveals previously unseen patterns and correlations [13]. Chronic heart disease (CHD), which restricts blood flow in the heart's arteries, is a leading cause of chest pain and stroke [14]. Chronic heart disease is a category of cardiovascular disease that affects the functionality of the heart over time and on a regular basis. It includes heart muscle disease (cardiomyopathy), congenital heart disease (CHD), pulmonary arterial hypertension, heart failure, arrhythmia (irregular heartbeat), coronary artery disease (CAD), and valve disease [15]. Table 1 presents its description.

**TABLE 1:** Description of chronic cardiac diseases.

Ref.	Cardiac disease	Description
[16]	Coronary Artery Disease (CAD)	Plaque accumulation restricts the coronary arteries, reducing blood supply to the heart and leading to chest pain (angina), dyspnoea, and potentially a heart attack. Coronary artery disease (CAD) is the most common chronic heart condition.
[17]	Congenital Heart Disease (CHD)	A congenital heart defect (CHD) is an anomaly in the structure of the heart or blood vessels. It may have an impact on blood flow, heart chambers, or valves. The severity of the defect affects the symptoms.
[18]	Heart Arrhythmia (Irregular Heartbeat)	An arrhythmia is a non‐normal cardiac rhythm that can result in symptoms such as palpitations, fainting, or dizziness. There are two types of arrhythmias: benign and malignant.
[19]	Heart Muscle Disease (Cardiomyopathy)	A disorder called cardiomyopathy impairs the heart muscle's ability to pump blood, weakening or stiffening it. Breathlessness, exhaustion, and heart failure could result from this.
[20]	Pulmonary Arterial Hypertension (PAH)	Elevated blood pressure in the arteries supplying the lungs with oxygen is known as PAH, or pulmonary arterial hypertension. This may result in exhaustion, chest discomfort, and dyspnoea.
[21]	Heart Valve Disease	Heart valve disease occurs due to malfunctioning cardiac valve or valves. This may result in a blockage of blood flow or a backflow of blood into the heart, which can induce exhaustion, breathing difficulties, and heart failure.
[22]	Heart Failure	A chronic disease known as heart failure (HF) causes the heart to weaken and become less effective at pumping blood throughout the body.

**FIGURE 2:**
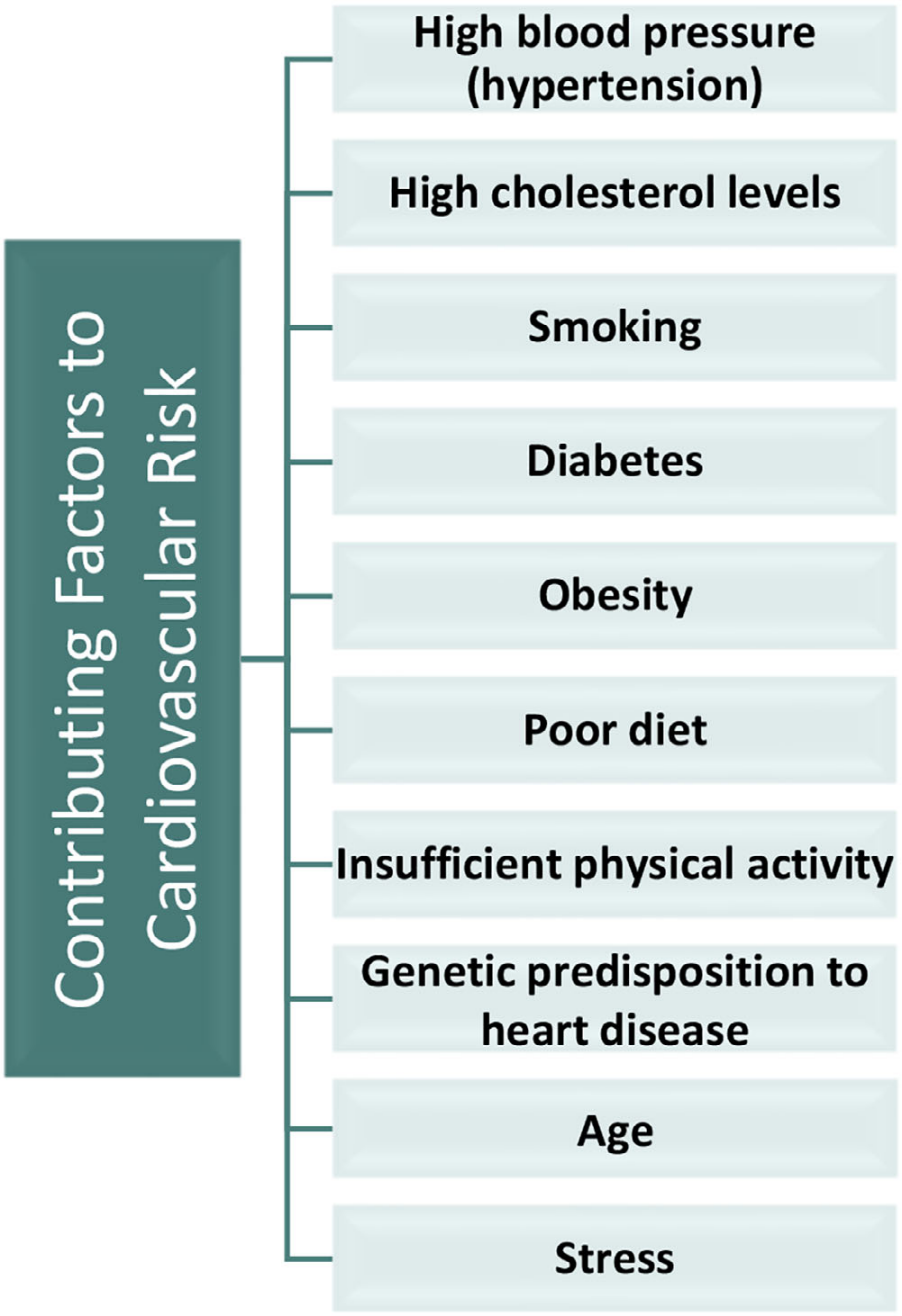
Contributing Factors to Cardiovascular Disease Risk.

Eighty percent of heart attacks cause symptoms such as vomiting, chills, dizziness, breathlessness, and unconsciousness, while 20 percent of heart attacks are silent and do not present any symptoms. Risk factors for cardiovascular disease include smoking, hypertension or high blood pressure, genetic predisposition, obesity, physical inactivity, diabetes, high cholesterol, alcohol use, existing heart conditions, and stress. It is anticipated that the number of heart disease patients may increase if adequate measures are not adopted. The transition to digital health presents healthcare professionals with ample opportunity to advance and increase the precision and accuracy of diagnosis [23]. The factors that elevate the risk of heart disease are presented in Figure 2.

Heart attack patients in the cardiac Intensive Care Unit (ICU) frequently experience complications that worsen their condition and increase their risk of death. Early detection of coronary heart disease (CHD) using machine learning algorithms can help reduce the mortality rate, making it crucial for patient survival. However, predicting CHD with these methods presents a significant challenge in medical data analysis [24].

Even though heart diseases are now the leading cause of death, they can still be properly treated and managed. How well a disease can be managed generally depends on when exactly it is diagnosed. Numerous scientists are employing statistics and data mining methodologies to assist in the identification of cardiac diseases [25].

Machine learning (ML), a branch of AI, aims to develop computer models capable of independently accessing and using data to learn on their own [26]. Through ML, systems can automatically evolve and learn from experience without needing explicit programming [27].

Deep learning is a subset of machine learning and artificial intelligence that mimics how people learn specific kinds of information [28, 29]. It is a crucial component of data science, which also involves predictive modelling and statistics [30]. Artificial intelligence, along with machine learning (ML) and deep learning (DL), is increasingly applied in predictive and design analysis for complex engineering challenges. These methods enhance the ability to analyse vast datasets, improve decision‐making processes, and optimise engineering solutions, demonstrating their significant impact across various fields [31]. One of several challenges in developing machine learning methods for early prediction of CHD is figuring out the best attributes to differentiate patients from the healthy population. In the field of computer science, the deployment of methods based on machine learning has become more significant in clinical decision‐making [32, 33].

**FIGURE 3:**
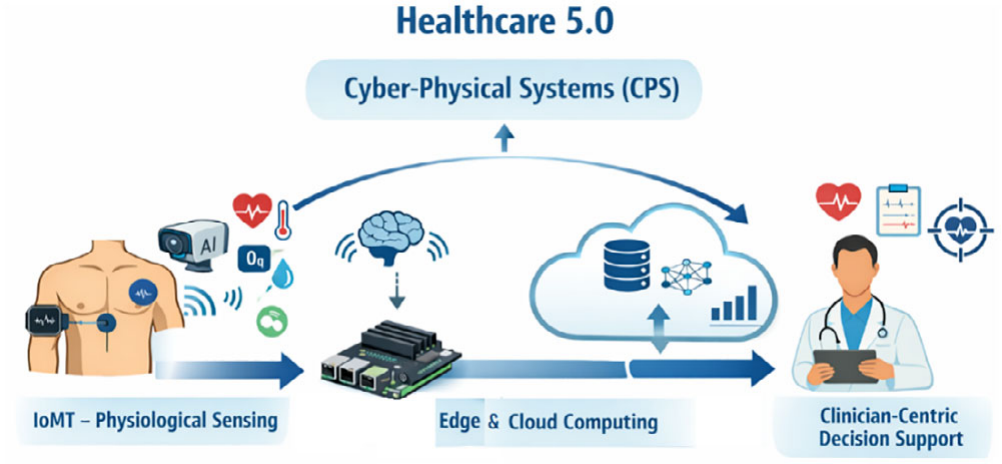
Link of healthcare 5.0 to the cyber‐physical system (CPS).

To improve decision‐making, physicians have recently relied more on digital tools. Machine learning (ML) is becoming a critical component in the healthcare sector, aiding in patient diagnostics [34]. Healthcare 5.0 represents a new wave of medical care, leveraging revolutionary technologies such as artificial intelligence (AI), the Internet of Medical Things (IoMT), and big data analytics to build intelligent, individualised, and efficient healthcare systems. By enabling real‐time monitoring, predictive analytics, and personalised treatment strategies, this shift aims to enhance patient outcomes [35]. By identifying patterns in the CHD dataset, machine learning techniques attempt to extrapolate the connection between the independent characteristics and the predicted outcomes. Thus, the advancement of machine learning algorithms allows for the early prediction of the risk of cardiac mortality. These techniques also assist medical professionals in making judgements that lead to an accurate diagnosis of heart diseases. Healthcare 5.0 is grounded in a human centred approach, where digital systems work alongside clinicians to support physical health processes in a continuous and responsive manner. Within this context, cyber‐physical systems, or CPS, enable the close interaction between medical data, computational intelligence, and clinical decision‐making. Edge cloud computing supports this structure by allowing time‐critical processing to occur near the data source and computationally intensive analysis and model refinement. IoMT serves as the data collection layer by facilitating the acquisition of physiological signals from connected medical devices. Through the integration of these components, the proposed framework is positioned as a Healthcare 5.0‐orientated system. Figure 3, represents the conceptual linking of Healthcare 5.0 to the cyber‐physical system (CPS).

In order to improve chronic disease prediction, this study investigates the combination of IoMT and deep learning, more especially convolutional neural networks (CNNs). The study illustrates how multimodal techniques can enhance early detection and diagnostic accuracy by focusing on ECG image categorisation and prediction of cardiac disease. The proposed study explores the integration of deep machine learning techniques in the IoMT‐enabled Healthcare 5.0 system for chronic disease prediction. It utilises convolutional neural networks to classify multimodal ECG images, improving accuracy and reliability. The findings suggest the potential for superior diagnostic performance in this framework.

Below is a list of the principal contributions.

- A multimodal CNN model for chronic cardiac disease prediction has been proposed. The suggested multimodal approach is integrated with IoMT sensors and offers a more precise means of diagnosing and treating chronic heart disease in healthcare 5.0.
- To enhance the generalisation and robustness of the model. It integrates two different ECG datasets.
- Extensive data preprocessing, including resizing, normalisation, and data augmentation techniques, has been applied.
- A K‐fold cross‐validation strategy has been employed to ensure model reliability and mitigate overfitting, providing a robust evaluation framework.

This is how the rest of the paper is structured. Section II provides a brief review of the literature. Section III explains the datasets and their descriptions. In Section IV, our proposed method, Multimodal for Chronic Cardiac Disease Prediction, is described. Section V includes results, comparisons, and discussions. Finally, conclusions and future studies are covered in Section VI.

## 2 Literature Review

The author [36] developed a deep neural network for the classification of 12 rhythm classes from 91,232 single‐lead ECGs from 53,877 patients. It is validated against an independent test dataset. DNN demonstrated an average area under the curve (AUC) of 0.97, which is high diagnostic performance, which is quite similar to the cardiologists. This approach reduced misdiagnosed computerised ECG interpretations and improved expert‐human interpretation efficiency.

In [37], a CNN model used by an AI‐enabled ECG is presented to identify atrial fibrillation during normal sinus rhythm. Positive patients had at least one ECG showing atrial fibrillation or atrial flutter. The test dataset was used to evaluate the performance of the proposed model, considering metrics such as accuracy, sensitivity, specificity, and F1 score, with an accuracy of 83.3%. In [38], various advanced machine learning algorithms were applied to achieve optimal performance for heart disease prediction. The algorithms employed included Random Forest, Support Vector Machine, Logistic Regression, and K‐Nearest neighbour classifiers. Logistic regression achieved the highest performance, with an accuracy of 91.80%, enhancing the identification of cardiac disease.

Ten machine learning classifiers across multiple categories were presented by the author in [39] to predict the risk of heart disease based on the full set of characteristics from the Cleveland heart dataset and the best attribute sets identified by three attribute assessors.

A 10‐fold cross‐validation testing option was used to evaluate the algorithms' performance. Additionally, the hyperparameter k, or the number of nearest neighbours for the instance‐based (IBk) classifier, was adjusted. Sequential Minimal Optimisation (SMO) with all collected features achieved an accuracy of 85.148%, with the highest accuracy reaching 86.468%. The author put forth a novel approach in [40] that seeks to improve the accuracy of cardiovascular disease prediction by identifying important features through the application of machine learning techniques. Using the hybrid random forest with a linear model (HRFLM), the proposed prediction model for heart disease, they achieved an accuracy level of 88.7%.

The objective of the author's study in [41] was to determine which data mining techniques and significant features could enhance the accuracy of cardiovascular disease prediction. Seven classification techniques were used to develop prediction models: k‐NN, Decision Tree, Naive Bayes, Logistic Regression (LR), Support Vector Machine (SVM), Neural Network, and Vote (a hybrid technique combining Naive Bayes and Logistic Regression). The experimental results indicate that the best‐performing technique, Vote, together with the identified significant features, could predict heart disease with an accuracy of 87.4%. In [42], the author assessed the potential of six machine learning techniques, including ANN, SVM, LR, k‐nearest neighbour (k‐NN), classification tree, and Naïve Bayes algorithms, for heart disease prediction. Eight different classification performance indices were used to evaluate these approaches, and the receiver operating characteristic (ROC) curve was applied to further assess their performance. The analysis demonstrated sensitivity and specificity of 89% and 81%, respectively, with LR achieving the highest classification accuracy of 85%.

A heart disease clinical decision support system based on rough sets‐based attribute reduction and the chaotic firefly algorithm (CFARS‐AR) was developed in [43] for the Statlog dataset. While the disease was being classified using the chaos firefly algorithm, the rough sets were utilised to minimise the amount of attributes. After that, the constructed model was contrasted with various models, including ANN, SVM, and NB. It showed an accuracy of 88.3%, and the suggested model performed the best out of all the models.

In [44], a comparative analysis was conducted on a hybrid model that was based on different machine learning models (LR, kNN, ANN, SVM, DT, NB, and RF) and feature selection approaches (relief, minimal‐redundancy‐maximal‐relevance (mRMR), and least absolute shrinkage and selection operator (LASSO)). Their investigation showed that the model's performance is impacted by the feature's removal. In comparison to other combinations employed in the study, the combination of Relief‐based feature selection and the LR‐based machine learning algorithm (MLA) yields superior accuracy up to 89.

The author in [45] utilised artificial neural networks to diagnose diseases, particularly heart disease, with high accuracy. A feed‐forward backpropagation neural network was employed to differentiate between the presence and absence of disease. The network consisted of thirteen input neurones, twenty hidden neurones, and one output neurone. Data from the UCI ML repository was used, and the network successfully classified 88% of the examples. In [46], the author explored the use of machine learning to analyse digital ECG data for diagnosing heart disease, employing techniques such as Random Forest, Support Vector Machine (SVM), and Logistic Regression. Additionally, heart‐related issues were predicted using Decision Tree Classifier models.

In [47], a dataset from the UCI repository consisting of 1,190 records was used to predict heart disease through five machine learning algorithms: Support Vector Machine, K‐nearest Neighbour, Naive Bayes, Ensemble Voting Classifier, and Logistic Regression. Before calculating accuracy, the relationships and correlations between attributes in the dataset were analysed, with Support Vector Machine achieving the highest accuracy at 85.49%, outperforming the other classification techniques. Finally, in [48], diseases were modelled using time‐domain components of the ECG signal, which were extracted with BIOPAC AcqKnowledge software. These helpful characteristics of the raw ECG signal can be used to identify cardiac arrhythmias. Heart rate, the QRS complex, the PR interval, the ST segment elevation, and the ST interval of the ECG signal are among the many ECG characteristics that were used in the analysis. These features of the ECG signal were used to diagnose a number of heart diseases, such as apnoea, myocardial infarction, sinus tachycardia, and atrial fibrillation. 85.7% was the highest accuracy that SVM recommended for a single person.

The author [49] considered vital signs such as heart rate, oxygen level, accelerometer, and ECG, as they have made early detection of chronic diseases possible. Nevertheless, current methods frequently rely on processing in the cloud, which raises latency and uses bandwidth. This study presented an accelerometer, temperature, and pulse rate sensor‐connected edge‐based Raspberry Pi heart disease prediction system. When the system was evaluated in several scenarios, its accuracy was 88%.

The primary aim of the study in [50] was to create effective disease prediction systems that could rapidly assess the severity of diseases like stroke and heart‐related conditions, predicting them with high accuracy. Patient parameters were initially categorised using a fog computing framework. A Deep Belief Network (DBN) combined with an Enhanced Grey‐Wolf Optimisation‐based Feature Selection Algorithm (EGWO‐FSA) was employed for heart and stroke prediction.

In [51], the authors proposed a cardiac learning model for predicting cardiac arrest survivability using sensors in the patient's body to detect heart rate variations. The model achieved management rates of 91.9% for heart rhythm, 88.5% for heart rate, 93.96% for cardiac arrest detection, 90.98% for anomalies, and 897.7% for supply monitoring. The author analysed ECG data from a cohort study of Chinese people in [52] using machine learning techniques. They used three one‐class classification approaches to identify anomalies and a multiclass strategy to predict the likelihood of hypertrophy, ischaemia, arrhythmia, and normalcy. The one‐class method has an accuracy of 75.6% and an AUC of 0.83 in identifying anomalies. With a 75.1% accuracy rate in four classes, the machine learning‐based classification indicates that cardiac abnormalities can be identified. Using data techniques to improve the dataset for the prediction of heart problems was advised by the author [53]. SVM provided an accuracy of 85%, which was superior and more useful. In SVM, sequential SVM performs worse than parallel SVM. Electrocardio gram topographies and a radial basis algorithm were employed in the suggested method in [54] to predict aberrant cardiac rhythm. Methods to predict ventricular arrhythmia, identify P‐QRS‐T waves, and find intervals in ECG signals were applied. Four techniques: feature extraction, QRS complex detection, ECG preprocessing, and classification were applied.

A multimodal framework for coronary computed tomography angiography (CCTA) prospective gating has been presented in [55], utilising seismocardiography (SCG) and electrocardiography (ECG). The approach produced tailored quiescence predictions for each cardiac cycle by adaptively fusing individual ECG and SCG‐based forecasts using a three‐layer ANN. When considering cardiac quiescence prediction, the fusion‐based approach outperformed the conventional ECG‐only method by 47%. In addition to increasing diagnostic quality, the fusion‐based prediction made CCTA gating more individualised and dependable.

In order to identify additional decline in critical care unit (CCU) patients at an early stage, the author predicted the short‐term prognosis of CCU patients in [56]. The CCU patients' ECG data (II, V3, V5, aVR induction) were transformed into picture data. A 2D CNN was utilised to predict the short‐term prognosis using the converted ECG pictures. 77.3% of the predictions were accurate. GradCAM's visualisation revealed that the CNN tended to highlight conditions like myocardial infarction and heart failure that were characterised by the shape and regularity of waveforms.

A multi‐label semi‐supervised model (ECGMatch) was suggested by the authors in [57] to identify several cardiovascular disorders at once with a minimal level of supervision. The model makes use of a label correlation alignment module, a hyper parameter‐efficient framework for pseudo‐label generation and refinement, and an ECG Augment module for the ECG data augmentation of both weak and strong.

In [58], the author put forth a brand‐new technique for converting photoplethysmography (PPG) to an electrocardiogram (ECG), turning it into a high‐quality source with no computational load. With an average correctness rate of 81%, the banded kernel ensemble technique predicts cardiac illnesses with exceptional precision. This approach might make it easier for healthy, high‐risk people to see doctors before things get too serious.

The study [59] leveraged a 12‐lead electrocardiogram (ECG) to provide a multi‐class classifier for predicting four types of cardiovascular diseases. Preprocessing, feature extraction, preparation, and augmentation of the data are all steps in the process. 16 time domain‐enhanced features are used by the classifier. Five classifiers were compared: Random Forest (RF), K Nearest Neighbours (KNN), Gradient Boost, Ada Boost, and XG Boost. Out of all these, XG Boost demonstrated the highest accuracy, at 93.0%.

The study aimed to enhance myocardial infarction (MI) risk assessment by combining factors related to cardiac troponin levels with patient age, sex, and timing. The myocardial‐ischaemic‐injury‐index (MI3), a machine learning model, was developed and validated on 7,998 patients after being trained on 3,013 individuals. MI3 uses gradient boosting to calculate the probability of an MI diagnosis, achieving 89.6% sensitivity and 89.3% specificity [60]. Another machine learning algorithm predicts the progression of hypertrophic cardiomyopathy (HCM) over ten years, forecasting six clinical variables with predictive regression models. Results showed the model's prediction error was lower than that of individual experts or expert panels [61].The study used ECG data from the ECG‐VIEW II database to offer an automatic diagnostic algorithm for cardiomyopathy and myocardial infarction. The model obtained 91.1% accuracy despite a small dataset and significant rates of misdiagnosis. For increased accuracy, researchers advise utilising 12‐channel ECG data or growing the dataset [62]. Global healthcare difficulties have been brought on by the COVID‐19 pandemic, with heart disease being the most common long‐term consequence. This study builds a deep neural network binary classifier for cardiac disease prediction using a preprocessed dataset of post‐COVID‐19 problems. It achieved an accuracy of 93.23% [63]. This proposed study combines machine learning and deep learning to develop a method for detecting chronic heart failure (CHF) based on heart sounds. It obtained a 92.9% overall accuracy rate. This might facilitate the process of identifying new CHF patients and facilitate the creation of CHF monitors that can be used at home to prevent hospital stays [64]. This paper reports on a study that used eleven machine learning classifiers to predict the existence of coronary heart disease. The researchers evaluated these algorithms' potential for heart disease prediction using a dataset from the UCI repository. The results demonstrated that Random Forest achieved the highest classification accuracy of 96.28% [65]. This work developed a computer‐aided diagnostic approach to identify congenital heart disease‐related pulmonary arterial hypertension (CHD‐PAH). It utilises cardiac cycle data extracted from pre‐processed heart sounds, convolutional neural networks (CNN), time‐frequency domain features, wavelet packet energy features, and double‐threshold adaptive segmentation. Classification into normal, CHD, and CHD‐PAH categories is achieved using XGBoost and a majority voting algorithm [66]. Additionally, a deep learning method, CHDdECG, is proposed for early congenital heart disease (CHD) detection using cost‐effective paediatric ECGs with human‐concept feature extraction, demonstrating paediatric ECGs’ broader diagnostic value [67].

Table 2 provides data types, methods, techniques, and limitations used by other researchers for cardiac disease prediction.

**TABLE 2:** Techniques, datatypes and limitations used by other researchers.

Ref	Method	Data type(s)	Limitations
[36]	DNN	ECG Signals	One of the study's shortcomings is that it used ambulatory monitor single‐lead ECG records, which might not have the same signal‐to‐noise ratio as 12‐lead ECGs.
[37]	Deep neural network	ECG	Because of ICD diagnostic codes and the inclusion of patients who had echocardiograms within 30 days, the study might have missed or misrepresented patients who had previously suffered from heart failure.
[38]	Random forest, support vector machine, logistic regression, and k‐nearest neighbour classifiers	ECG	Because the study only uses one dataset, its generalisability is constrained.
[39]	Random forest classifier, support vector machine classifier, logistic regression classifier, and K‐nearest neighbour classifier	—	The limited size of the study's dataset might make it more difficult to extrapolate results to bigger and more varied populations, which could have an impact on how reliable the predictive models are.
[40]	Hybrid random forest	EHR	The Cleveland dataset from the UCI repository is the only dataset used in the study, which limits its generalisability.
[41]	K‐NN, decision tree, naive Bayes, logistic regression (LR), support vector machine (SVM), neural network, and vote	EHR	The Cleveland dataset from the UCI repository is the only dataset used in the study, which limits its generalisability.
[42]	ANN, SVM, LR, k‐nearest neighbour (KNN), classification tree and NB	EHR	EHR data quality and completeness can affect predictive models for heart disease, potentially leading to biased predictions or reduced accuracy due to inaccuracies, missing data, or inconsistencies.
[43]	Hybrid model fuzzy clustering	EHR	The quality and representativeness of the input dataset, which may be limited if it is not diverse or representative of various demographic groups or clinical situations, are critical to the system's effectiveness.
[44]	LR, KNN, ANN, SVM, DT, NB, and RF	EHR	Despite its popularity, the Cleveland heart disease dataset may be too dependent on it, limiting its generalisability to different clinical settings and demographics and perhaps missing important clinical signs or adding biases that impact diagnostic outcomes.
[45]	ANN	EHR	The Cleveland dataset from the UCI repository is the only dataset used in the study, which limits its generalisability.
[46]	Random forest, support vector machine (SVM), and logistic regression	ECG	Handcrafted features
[47]	Support vector machine, logistic regression, K‐nearest neighbour, naive Bayes, and ensemble voting classifier	ECG	Low Accuracy
[48]	Support vector machine	ECG	Low Accuracy
[49]	Naive Bayes, support vector machine (SVM), decision tree, and K‐nearest neighbour (KNN)	ECG	When it comes to computing power, devices like Raspberry Pi might not be as powerful as cloud‐based solutions.
[50]	Deep belief network (DBN) enhanced grey‐wolf optimisation‐based feature selection algorithm (EGWO‐FSA).	ECG	The significant gaps of excessive energy usage and lengthy processing times need to be addressed in the suggested framework.
[51]	Deep cardiac learning algorithm	ECG	Although the sensors offer continuous monitoring, not all patients may be a good fit for their intrusive implantation.
[52]	ML Algorithm	ECG	PPG and ECG waveforms provide breathing data, the quality of which is greatly patient‐dependent
[53]	SVM and Sequential SVM	—	The quality and representativeness of the input dataset, may be limited The SVM algorithms and the overall model's performance are significantly influenced by the quality of the data.
[54]	PAT algorithm	ECG	The proposed method for prediction in real‐time, but may introduce delays, particularly in emergencies requiring immediate detection and response.
[55]	ANN	ECG	The interventricular septam (IVS) motion might not precisely reflect the motion of coronary arteries.
[56]	CNN	ECG	The study's findings are based on a particular patient cohort in a single critical care unit; hence, for wide use, more validation in various clinical contexts is required
[57]	Semi‐supervised model (ECGMatch)	ECG	This method involves complex modules like information distillation and ECG Augment, which could demand a large amount of training time and computer resources.
[58]	Banded kernel ensemble technique	ECG	Because of the complexity and separation of the data sources, integrating waveform data with disease codes from databases such as MIMIC III can be difficult and calls for more automated and dependable processing techniques.
[59]	Random Forest (RF), K Nearest Neighbours (KNN), Gradient Boost, Ada Boost, and XG Boost	ECG	This method requires the patient or user to wear a Holter monitor to continuously monitor CVD.
[60]	Myocardial Ischemic Injury Index (MI3)	EHR	It considers limited variables and a specific time frame.
[61]	Machine learning and SHAP method for Hypertrophic Cardiomyopathy prediction	EHR	The suggested study is based on data from a single medical centre
[62]	CNN for MI and Cardiomyopathy prediction	ECG	Because the study only uses one dataset, its generalisability is constrained.
[63]	Stacking ensemble‐based binary classifier, DNN	EHR	The model's generalisability is limited because it was created especially to predict cardiac disease in COVID‐19 survivors.
[64]	Deep Learning and classic Machine Learning	PCG signals	Differences in PCG audio recordings made from different body positions may cause data discrepancies that could affect the model's functionality.
[65]	Random forest and Gradient‐boosting tree	EHR	The dataset is rather limited and might not accurately reflect the variety of heart disease cases, were used in the study.
[66]	CNN	Heart Sound Data	The dataset is collected from individuals who were already informed; size and diversity, however, seem to be restricted.
[67]	Deep learning‐based model, CHDdECG	ECG	The model uses limited features, which may limit its ability to capture all important patterns.

Table 2 provides an overview of existing ECG‐based cardiac disease prediction studies, summarising the deep learning techniques employed, the types of data which have been utilised, and the associated methodological limitations. It can be observed that most of the studies rely on a single limited dataset, limited variables, and complex modules. These works predominantly apply conventional deep neural networks or CNN, random forest, DNN, SVM, and ANN. Common limitations reported across prior works include the use of single‐lead ECG recordings, limited‐sized datasets, restricted signal diversity, and lack of validation. These constraints highlight the challenges which are related to signal quality, generalisability, and clinical robustness. These all motivate the need for more reliable and scalable intelligent ECG analysis frameworks.

## 3 Methodology

**FIGURE 4:**
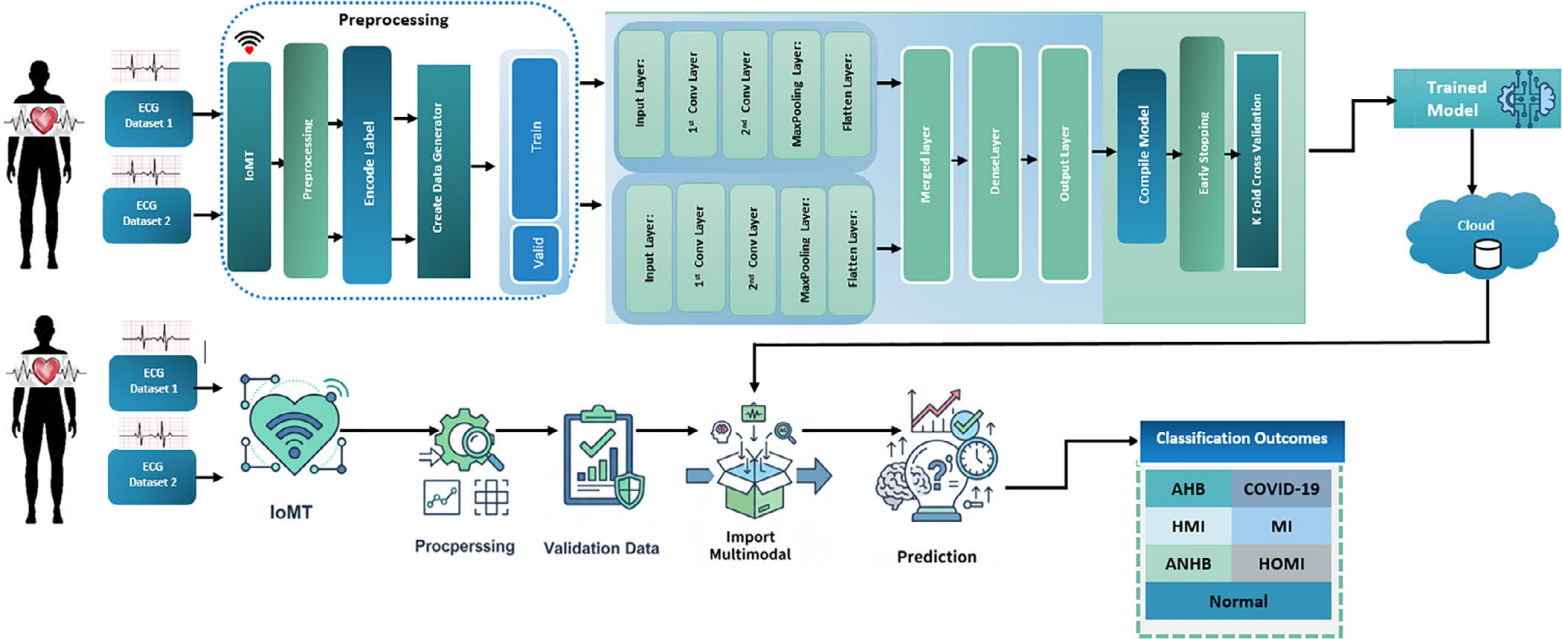
Multimodal CNN for chronic cardiac disease prediction.

The flowchart of the proposed model, as depicted in Figure 4, is broken down into multiple stages: (1) data gathering via IoMT devices; (2) preprocessing of raw data; and (3) data distribution for training and validation within the proposed IoMT‐enabled CNN‐based multimodal edge cloud layer, which includes the storage of the trained model and the validation layer. This study utilises two different electrocardiogram (ECG) image datasets to diagnose chronic cardiac disease using a multimodal deep learning technique. The sensory layer used for data collection comprises sensors and IoMT devices. The methodology involves several key steps to develop a multimodal deep machine learning model for predicting heart disease using two ECG datasets. Each dataset undergoes preprocessing, where images are resized to a standardised (128, 128) pixel format and normalised. Labels corresponding to the image classes are encoded and converted into a categorical format for classification tasks. The datasets are then split into training and validation sets, ensuring balanced distribution across these subsets. The multimodal model architecture is built using two separate convolutional neural networks (CNNs), one for each dataset. These networks shared merged layers & dense layers and concatenated their outputs after sharing the first few layers for feature extraction. The compilation of the proposed model is performed with the Adam optimiser and categorical cross entropy loss function, and the process of training includes early stopping based on validation loss. Cross‐validation with K‐fold validation effectively assesses model performance across multiple data subsets, offering a more reliable gauge of generalisability. While an initial data split establishes training and validation sets, K‐fold cross‐validation enhances this by systematically alternating distinct subsets for training and validation. This method improves the multimodal CNN model's robustness and dependability in classifying heart disease by expanding the evaluation across different data samples. Multimodal is stored on the cloud. In order to guarantee low latency and effective predictions, which are essential for healthcare applications, edge computing is given priority in our architecture for on‐site data collection, preprocessing, and real‐time inference. The present study concentrates on model training and validation carried out in a cloud‐based environment (Google Colab) for computational efficiency, but the cloud layer is a future extension for scalable data storage, model maintenance, and advanced analytics. While the cloud facilitates long‐term data handling and system updates, the trained models are ultimately meant to be deployed on edge devices to enable fast and localised disease prediction, striking a balance between immediate responsiveness and scalability and maintainability. The model is imported from the cloud, validated, and a prediction is made; it predicts the categories of chronic cardiac disease, and then its predictive power is evaluated.

Finally, the model's performance metrics, including accuracy and loss, are evaluated. The accuracy metric, Adam optimiser, and categorical cross‐entropy loss were used in the compilation of the model. Early pausing was used in K‐fold cross‐validation with 5 folds to avoid overfitting during training. Its adoption is a purposeful and essential methodological decision. In particular, K‐fold cross‐validation is a crucial technique for guaranteeing robust model performance because of the variability and potential imbalance in healthcare data, such as variations in patient populations and disease prevalence. This approach reduces overfitting. It improves the model's generalisability by methodically training and validating across numerous folds.

Two publicly accessible and clinically relevant datasets, from Mendeley and Kaggle, are used in this study to classify chronic cardiac disease using multimodal ECG data. Rather than participant‐level collection, publicly available datasets were utilised, as the IoMT component is considered as a conceptual deployment framework. These datasets were chosen because they provide a representative baseline for assessing our suggested multimodal CNN model because of their varied labelling processes and unique acquisition settings.

Stratified data splitting, balanced class representation, and thorough performance evaluation across various measures are some of the tactics used to assure robust model building, even if we acknowledge the small size and institutional scope of these datasets. Furthermore, the multimodal CNN architecture was created to be generalisable and flexible, allowing for a smooth transition to bigger or institution‐specific datasets in subsequent implementations.

To provide insights into model performance across various folds, evaluation measures such as accuracy were calculated post‐training, along with visuals showing fold‐wise training and validation accuracies. This strategy ensures that the development of reliable deep learning models in biomedical research settings follows an organised and efficient process. The training utilised 80% of the data, with the remaining 20% allocated for validation, ensuring a comprehensive assessment of the model's predictive accuracy for heart disease classification tasks. Although accurately predicting human diseases is a challenging endeavour in the pursuit of better and faster treatments [68, 69, 70], machine learning techniques are employed to bridge any gaps in the fields of medical image processing and other domains for the analysis and forecasting of medical image features. Additionally, deep learning is essential for the successful early detection of medical conditions [71, 72]. Early disease detection and treatment reduce the chance of the disease getting worse and, as a result, raise linked mortality [73, 74, 75, 76, 77]. Performance of the proposed multimodal shows that it'll be really helpful to reduce this rate. Table 3 presents a description of variables and symbols used in the multimodal algorithm, and Table 4 presents the multimodal algorithm.

**TABLE 3:** Variables and symbols for the multimodal algorithm.

	Descriptions
ECG1_images	Images from the first ECG Dataset.
ECG2_images:	Images from the second ECG Dataset.
C:	Preprocessing, model training, and cross‐validation are examples of configuration options.
Preprocessing_completed:	Boolean variable to monitor the completion of preprocessing.
Model_training_completed:	Boolean variable to monitor the completion of Model Training
Cross_validation_completed	Boolean variable to monitor the completion of cross‐validation

**TABLE 4:** Algorithm of multimodal for chronic cardiac disease prediction.

Algorithm: Multimodal chronic cardiac disease prediction
**Input**:
‐ ECG Dataset 1 images
‐ ECG Dataset 2 images
‐ Configuration options C = {preprocessing, model_training, cross_validation}
**Output**:
‐ Predicted labels for each test image
**BEGIN**
**if C includes preprocessing then**
Resize and normalise ECG Dataset 1 and ECG Dataset 2 images
Encode labels
Split data into training and validation sets
**end if**
**if C includes model_training then**
Define a multimodal CNN model integrating both datasets
Compile the model with optimiser and loss function
Train the model with early stopping
**end if**
**if C includes cross_validation then**
Perform K‐Fold cross‐validation
Record training and validation accuracies
**end if**
Initialize an empty list for predicted labels
for each test image pair do
Predict labels using the trained multimodal model
Add predicted labels to results
**end for**
return Predicted labels
**END**

A CPU‐only environment on Google Colab was used to quantify the inference time in order to assess the model's potential for real‐time deployment. Prediction scenarios were simulated using 100 randomly generated ECG data, each measuring 128 × 128 × 1. An average of 37.92 milliseconds was found to be required for a single forecast. This research shows that the suggested multimodal CNN model can effectively produce predictions under real‐time restrictions, which qualifies it for incorporation into edge computing systems offered by IoMT. It is mentioned that using cloud‐based or GPU‐enabled platforms can further cut down on this inference time, improving responsiveness in real‐world clinical settings.

### 3.1 Multimodal Fusion Formulation

The proposed framework adopts **early fusion at the feature level**. Two parallel CNN branches independently process ECG dataset 1 and ECG dataset 2 image modalities; multimodal fusion is implemented using an **early which is feature‐level fusion strategy**. Let $X^{\left(1\right)}$ and $X^{\left(2\right)}$ denote the two ECG image modalities. Each modality is processed through an independent CNN branch to extract feature vectors *f_1_* and *f_2_* respectively. These feature representations are concatenated to form a joint feature vector: [*f_1_* ||*f_2_*]

The concatenation operation is indicated by ||. A softmax classifier is used to make the final prediction after the fused feature vector has been passed through fully connected layers. When compared to late fusion, which combines decisions from different modalities only at the output level, early fusion allows the network to acquire intermodality correlations during training, which improves discriminative capabilities.

### 3.2 Computational Complexity Derivation and Analysis

Convolutional and fully linked layers make up the majority of the suggested multimodal CNN model. We calculate the computational complexity for these crucial elements below:

$$FLOPs_{conv}=H_{o}\;\times \;W_{o}\;\times C_{out}\;\times K^{2}\;\times \;C_{in}\;\times 2$$

$H_{o},W_{o}\;:\;$ Height and width of the output feature map

$C_{in},C_{out}\;:\;$ Number of the input and output channels

**K**: Size of kernel assumed squares

One addition and one multiplication per operation are taken into consideration by factor 2. Table 5 depicts the summary of calculations of complexity.

**TABLE 5:** Complexity calculation summary.

Layer	Input size	Output size	FLOPs (multiply + add)
Conv Layer 1 (Branch 1)	128 × 128 × 1	126 × 126 × 32	9.18 million
Conv Layer 1 (Branch 2)	128 × 128 × 1	126 × 126 × 32	9.18 million
Fully Connected Layer 1	508,032	64	65.03 million
Fully Connected Layer 2	64	32	4,096
Total FLOPs			∼83.4 million FLOPs

The computational cost of the suggested multimodal CNN model is 83.4 million FLOPs, with thick layers predominating because of the high volume of input characteristics. Despite their contribution, convolutional layers are still less costly. This complexity balances computational efficiency and prediction accuracy, making it appropriate for deep learning models in healthcare imaging inside IoMT‐enabled Healthcare 5.0 systems. Large, fully connected layers in the model, however, could result in increased power consumption or latency. The optimisation of the architecture to lower computing overhead without compromising accuracy may be the main focus of future research.

## 4 Dataset

Many earlier studies over the past ten years have demonstrated the potential and viability of using ECG signals to identify a broad range of cardiovascular diseases (CVDs) [78, 79, 80, 81, 82]. With the rapid growth of deep learning techniques, numerous researchers have employed end‐to‐end deep learning models to generate accurate CVD predictions. This study utilised two different datasets obtained from online repositories, Kaggle and Mendeley. Although both datasets include ECG images, they represent different modalities due to variations in sources, patient demographics, data collection techniques, and clinical situations. Each dataset provides distinct diagnostic data. There are four categories in the second dataset and five in the first. Two of the categories are similar; however, there are additional distinct categories that show distinct cardiac problems.

A wide range of cardiac disorders is included in the dataset, many of which are correlated, either directly or indirectly, with chronic heart problems. For example, underlying cardiac diseases such as coronary artery disease or cardiomyopathies, which are frequently chronic in nature, might result in atrioventricular heart block (AHB). Heart symptoms associated with COVID‐19 underscore the systemic influence of the virus on the circulatory system, perhaps aggravating pre‐existing chronic heart problems or initiating new ones. Thickening of the heart muscle causes hypertrophic cardiomyopathy (HMI), a chronic hereditary condition that alters the structure and function of the heart over time. Heart attacks, sometimes referred to as myocardial infarctions (MI), are often linked to long‐term health issues such as diabetes, hypertension, and atherosclerosis, which put people at risk for acute cardiovascular events. Insights into disease progression, risk assessment, and therapeutic management approaches for individuals with chronic cardiac conditions can be gained by understanding and evaluating these conditions within the dataset.

**Atrioventricular Heart Block (AHB)**: This condition is characterised by a delay or interruption of electrical signals between the upper chambers (atria) and lower chambers (ventricles) of heart. This may result in abnormal cardiac rhythms and reduced heart rates.

**Myocardial Infarction (MI)**: Prolonged coronary artery disease is a common cause of MI, which requires prompt diagnosis and treatment to provide optimal patient outcomes.

**Hypertrophic Cardiomyopathy (HMI)**: It is generally considered a chronic condition. The thickening of the heart muscle, especially in the left ventricle. It is a hereditary condition. Over time, this thickness may cause a number of symptoms and consequences, including arrhythmias, chest discomfort, shortness of breath, and an elevated risk of sudden cardiac mortality.

**COVID‐19**: The virus can cause myocarditis and other chronic cardiac diseases that can be tracked by changes in the electrocardiogram.

**Abnormal Heart Beat (ANHB)**: Patterns of abnormal heartbeats can be used to diagnose chronic arrhythmias. Chronic diseases that require for ongoing monitoring might result from persistent arrhythmias.

**History of Myocardial Infarction (HOMI)**: It describes a previous instance of a myocardial infarction, indicating that there has been injury or death to the affected tissue due to a blockage of blood flow to a portion of the heart muscle. To prevent further complications, a history of myocardial infarction requires ongoing medical therapy, as it is a significant risk factor for future heart‐related issues.

**Normal**: A control group that helps distinguish between healthy and diseased states.

### 4.1 Dataset 1

First Dataset “heart_disease_prediction_image_dataset” is taken from Kaggle [83]. It consists of ECG images of AHB (Atrioventricular Heart Block), COVID‐19, HMI (Hypertrophic Cardiomyopathy), MI (Myocardial Infarction), and NORMAL (ECG). It consists of ECG images, each of which is a 640 × 640 JPG. Table 6 represents the number of instances and categories of dataset 1.

**TABLE 6:** Dataset 1 number of instances.

Category	Instances
AHB	516
COVID‐19	238
HMI	185
MI	71
Normal	816

**FIGURE 5:**
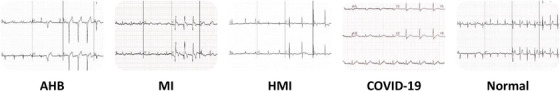
ECG Images from Dataset 1 labelled by category.

Figure 5 represents ECG images from the first dataset.

### 4.2 Dataset 2

The second dataset “ECG Images dataset of Cardiac Patients”, is taken from Mendeley [84] which is an online data repository. It consists of ECG images of abnormal heart beat, myocardial infraction, history of myocardial infraction (HMI), and normal. Each ECG image is 2213 × 1572 jpg. Table 7 represents the number of instances and categories of dataset 2.

**TABLE 7:** Dataset 2 number of instances.

Category	Instances
ANHB	93
HOMI	69
MI	96
Normal	114

**FIGURE 6:**
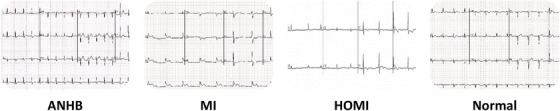
ECG images from dataset 2 labelled by category.

Figure 6 represents ECG images from the first dataset.

The significance of multimodal learning is highlighted in the given multimodal approach for chronic cardiac disease prediction.

## 5 Dataset Preprocessing

**FIGURE 7:**
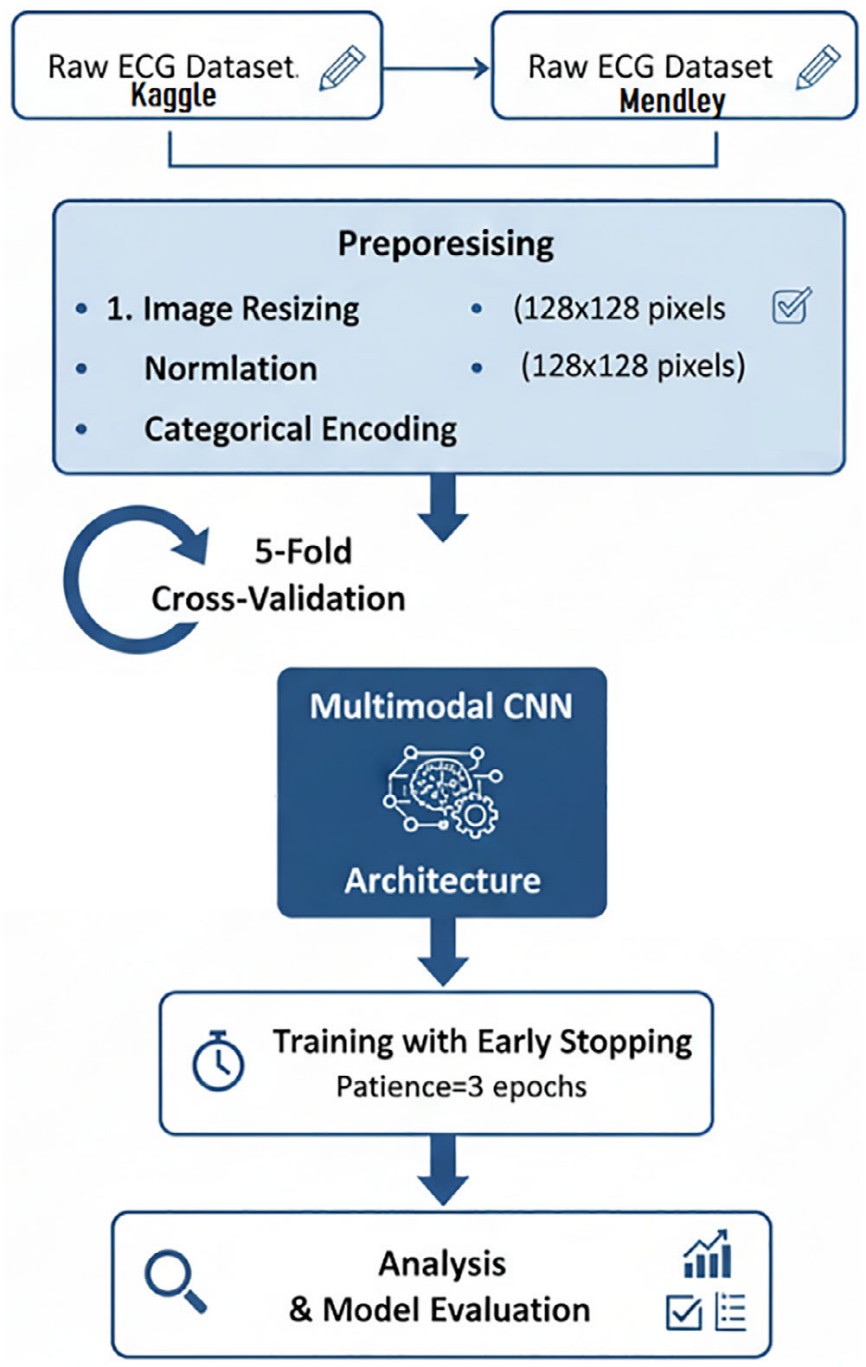
ECG analysis workflow.

As two publicly accessible ECG image datasets were obtained from Kaggle and Mendeley, where Institutional Review Board (IRB) approval was not necessary because both datasets were pre‐anonymised and all patient‐identifiable information had already been eliminated, both of these were utilised in this study, and all the images from both of the datasets were resized to 128 × 128 pixels to make them uniform, and then these all were normalised to ensure consistent input scales for the convolutional neural networks (CNNs). The class labels were encoded and transformed into a categorical format, which is suitable for multi‐class classification. To maintain a balanced representation of each class, the datasets were then divided into training and validation sets, with 20% of the data reserved for validation. Additionally, 5‐fold cross‐validation was employed, which helps to assess the model's generalisability and its robustness. Each dataset was initially processed through a dedicated CNN. The ECG preprocessing workflow is shown in Figure 7.

## 6 Result and Discussion

The significance of multimodal learning is highlighted in the given multimodal approach for chronic cardiac disease prediction, where both modalities are based on ECG images. A more robust and accurate classification of cardiac abnormalities can be achieved by integrating different perspectives or representations of ECG images. It encompasses variations in imaging techniques, image quality, and contextual information, enabling richer and more comprehensive feature extraction. By combining different modalities, a wider range of data can be captured, revealing subtle patterns that may not be apparent when examining each modality in isolation. Furthermore, by learning to identify common patterns across various representations of ECG images, the model can enhance its generalisation capabilities and perform better on unknown or heterogeneous data.

**FIGURE 8:**
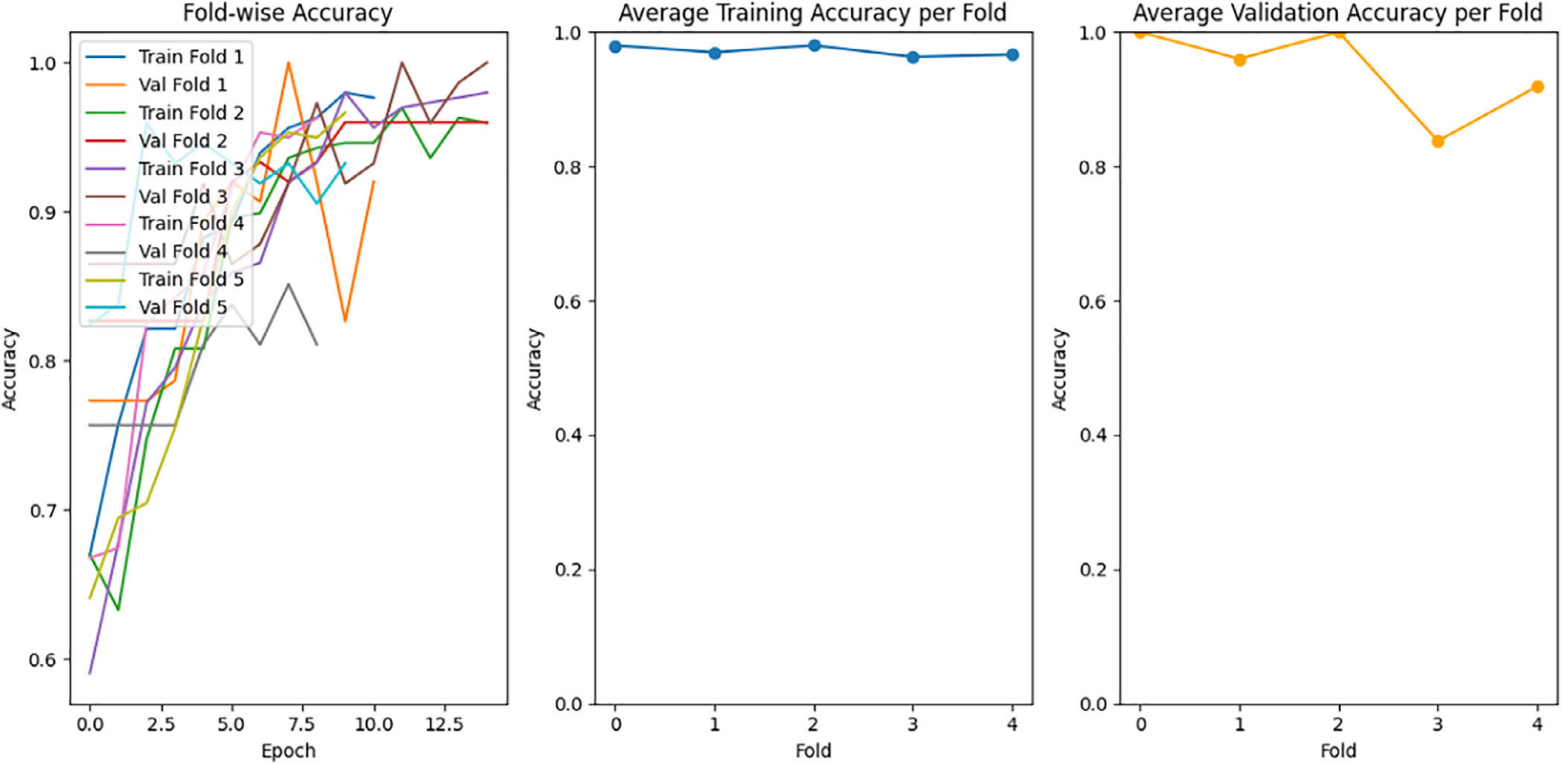
Validation and training accuracy of the proposed model across 5 folds.

Figure 8 illustrates the fold‐wise accuracy and calculates the average accuracy per fold, along with the average validation accuracy. Images from electrocardiograms (ECGs) from both datasets have been used to train and assess the multimodal model for chronic cardiac disease classification. Across several training runs, the multimodal model for heart disease prediction based on the two ECG datasets has demonstrated promising performance. The model has demonstrated an average high training accuracy of 97.18% and a validation accuracy of 94.34%. These findings demonstrate that the model is effectively generalising to previously unseen validation data while learning from the training dataset. Notably, consistently high validation accuracy has been achieved, indicating robust and reliable predictions, which underscores its significant generalisation potential beyond the training and validation sets. Overall, the model has exhibited strong performance metrics, suggesting its applicability in predicting cardiac disease using multimodal ECG data.

**FIGURE 9:**
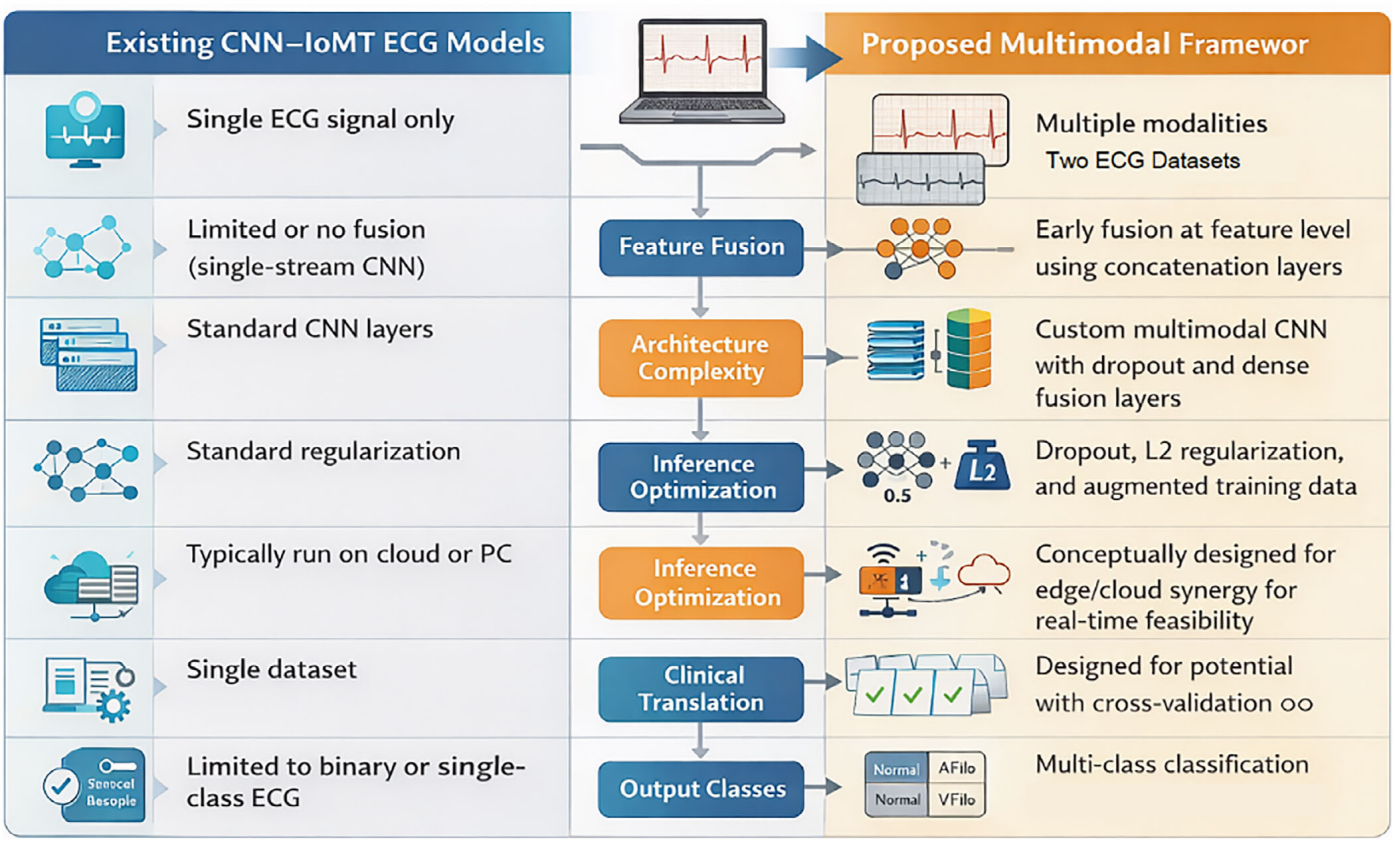
Comparison with existing IoMT‐CNN models.

Figure 9 provides a conceptual comparison; the “Existing CNN–IoMT ECG Models” column lists common features of traditional single‐modality CNN–IoMT ECG systems. Through multimodal fusion, enhanced regularisation, and possible real‐time deployment viability, the suggested framework exhibits innovation.

Some limitations of this approach include the large dataset size, diversity, and potential issues with data consistency and quality. Additionally, it faces challenges in clinical settings, particularly when interpreting decisions made by complex deep learning models, necessitating thorough clinical validation with medical specialists.

### 6.1 Tools and Environment

The multimodal deep learning model, which integrated two ECG datasets, was implemented and trained using Google Colab. This platform provided essential computing resources for the creation and evaluation of models, seamlessly integrating with TensorFlow. The performance of the proposed IoMT‐enabled model across multiple modalities was visualised and analysed using Google Colab, highlighting its suitability for complex multimodal data processing in healthcare applications. Table 8 presents the details of the tools and devices used. The model's predictive performance can be evaluated using the confusion matrix.

**TABLE 8:** Tool and environment.

Device specification	Description
System	DESKTOP‐OEA1KN4
Processor	Inter Core i7‐7700 HQ CPU @ 2.80 GHz 2.80GHz
RAM	16 GB
Google Colab Version	Python 3.10.12

Table 9 lists other training‐related characteristics and training preferences. These training options and parameters yielded the most effective outcomes in this study after being validated under various conditions.

**TABLE 9:** Training settings and parameters.

Training preferences	Values
Size of image	128 × 128
Total number of epochs	15
Iterations within each epoch	500
Total iterations	7500
Starting learning rate	0.001
Execution environment	Google Colab
Minibatch size	16
Shuffle	Per epoch
Early stopping patience	3 epochs
K‐fold cross‐validation	5 folds
Validation split	20% of data

**FIGURE 10:**
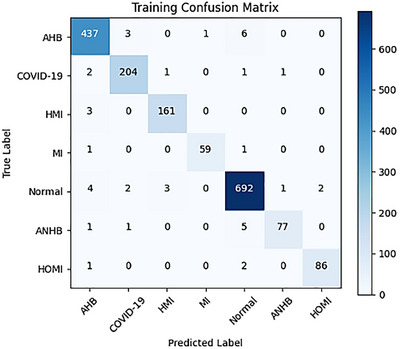
Confusion matrix of training.

The confusion matrix of training and validation comprises two categories to analyse performance, including accuracy, as Figure 9 and Figure 10 demonstrate.

Figure 10 represents the confusion matrix of training of the suggested model. An extensive summary of the model's performance across seven different classes of cardiac diseases is presented in the training confusion matrix. Although it correctly identified 437 cases of AHB, there were sporadic misclassifications, including 3 cases that were correctly identified as COVID‐19, 1 case as myocardial infarction, and 6 cases as normal. It is noteworthy that it did not misclassify ANHB and HOMI in its predictions. With 204 cases, the model produced appropriate predictions for COVID‐19; however, it incorrectly identified 2 cases as AHB, 1 as an HMI, and 1 as ANHB. Similarly, HMI showed no mistakes in predicting COVID‐19, MI, Normal, ANHB, or HOMI, properly identifying 161 cases and having 3 misclassifications as AHB. The model correctly identified 59 cases of MI but incorrectly classified 1 as AHB and 1 as Normal. With 692 correct predictions, it did a good job of detecting Normal cases, but it misclassified 4 as AHB, 2 as COVID‐19, 3 as HMI, 1 as ANHB, and 2 as HOMI. The model successfully identified 77 cases of ANHB, but it incorrectly identified 1 as AHB, 1 as COVID‐19, and 5 as Normal. Finally, in terms of HOMI prediction, it detected 86 occurrences accurately but incorrectly classified 2 as AHB, 2 as Normal, and 1 as ANHB. This in‐depth review highlights the model's advantages as well as potential areas for improvement in terms of how well it can differentiate between various chronic cardiovascular disorder categories.

**FIGURE 11:**
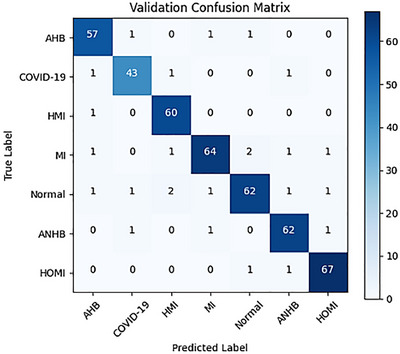
Confusion matrix of validation.

An informative perspective on the model's performance on validation data from seven different classes of heart disease is provided in Figure 11 by the validation confusion matrix. The validation confusion matrix provides information on how well the model performs for each of the seven different kinds of cardiac conditions. It predicted 57 cases of AHB with accuracy and just a few minor misclassifications; 1 case each of COVID‐19 and MI was incorrectly classified. The model produced 43 accurate predictions for COVID‐19, with 1 misclassification of AHB and 1 for ANHB. It accurately detected 60 examples in the HMI prediction without making any mistakes in the other categories. Comparably, the model for MI accurately predicted 64 instances but misclassified 1 as AHB, 2 as Normal, and 1 as COVID‐19, ANHB, and HOMI, respectively. Although it correctly recognised 62 cases of Normal, it incorrectly identified 1 as AHB, COVID‐19, HMI, ANHB, and HOMI. The model successfully predicted 62 cases of atrial non‐heart block (ANHB) and misclassified 1 case each of COVID‐19, Normal, and HOMI. Last but not least, it accurately detected 67 cases of HOMI and incorrectly classified 1 case as each of Normal and ANHB. This analysis identifies the model's strong points and potential areas for improvement in terms of accuracy in differentiating between these various kinds of cardiac disorders.

The precision, recall, specificity, and F1‐score metrics provide a range of statistical measurements for performance and comparison. Table 10 represents the performance metrics of the study, including accuracy, precision, recall, specificity and F1 score. These parameters are computed using the formulas in Equations (1) to (5), as indicated below [81].

(1) $$\mathrm{Accuracy}\;=\frac{TP+TN}{TP+TN+FP+FN}\;$$

(2) $$\mathrm{Precision}=\frac{TP}{TP+FP}\;$$

(3) $$\mathrm{Recall}\;=\frac{TP}{TP+FN}$$

(4) $$\mathrm{Specificity}=\frac{TN}{TN+FP}\;$$

(5) $$F1\;\mathrm{Score}=2\times \frac{\mathrm{Precision}\;\times \;\mathrm{Recall}}{\mathrm{Precision}+\mathrm{Recall}}$$

**TABLE 10:** Performance of multimodal.

Class	Precision	Recall	Specificity	F1 score	Accuracy
**Training results**					
AHB	0.97	0.978	0.998	0.974	0.978
COVID‐19	0.976	0.986	0.998	0.981	0.976
HMI	0.981	0.981	0.999	0.981	0.981
MI	0.967	0.935	0.999	0.951	0.967
Normal	0.983	0.987	0.996	0.985	0.983
ANHB	0.97	0.917	0.999	0.943	0.917
HOMI	0.977	0.966	0.999	0.971	0.966

Class	Precision	Recall	Specificity	F1 score	Accuracy
**Validation results**					
AHB	0.931	0.95	0.997	0.94	0.95
COVID‐19	0.935	0.924	0.998	0.93	0.935
HMI	0.944	0.984	0.997	0.964	0.984
MI	0.96	0.914	0.997	0.936	0.914
Normal	0.938	0.899	0.998	0.918	0.899
ANHB	0.945	0.954	0.998	0.949	0.954
HOMI	0.962	0.971	0.998	0.966	0.971

**FIGURE 12:**
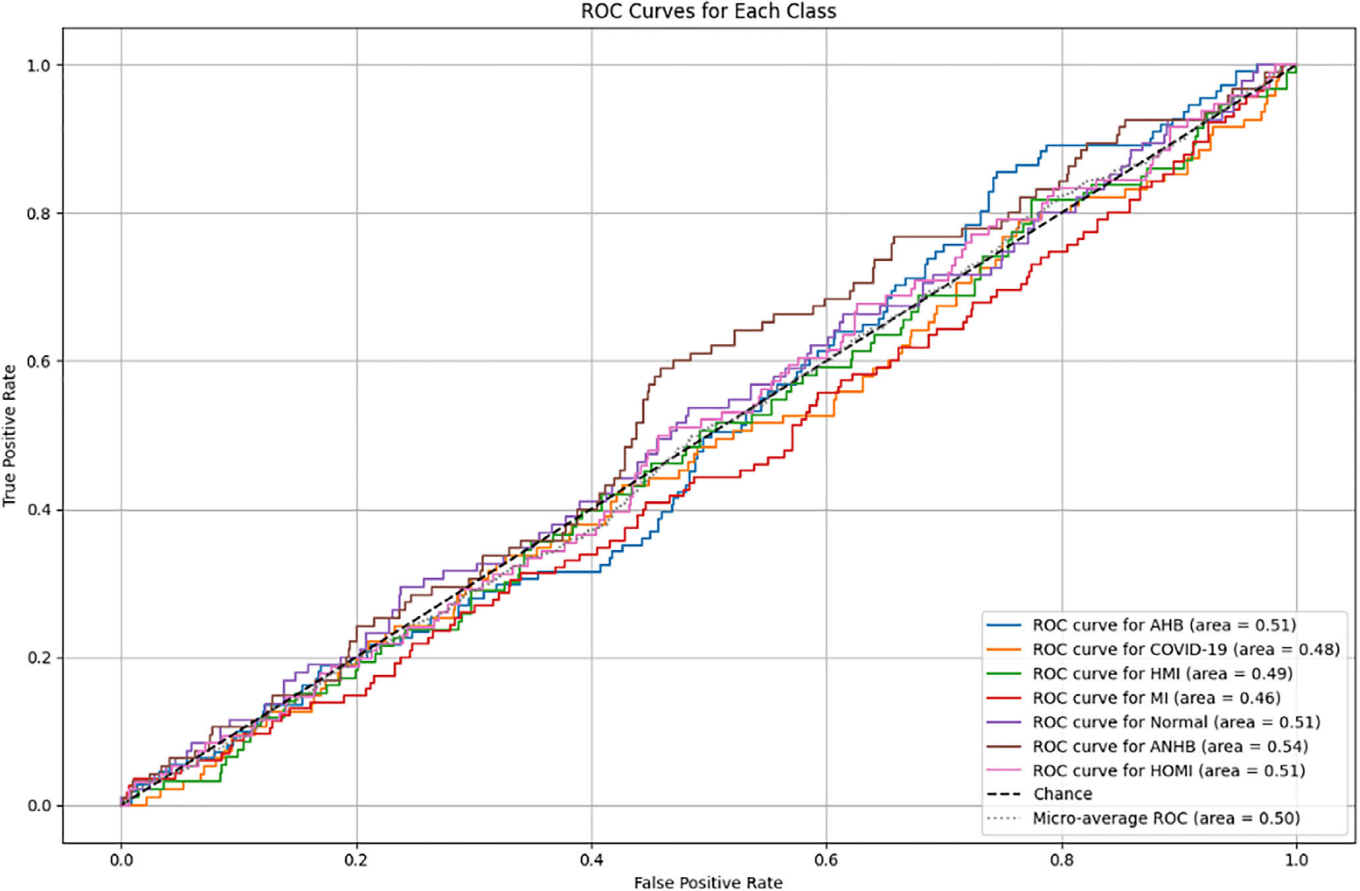
ROC curves for each class.

Class‐wise Precision‐Recall (PR) and Receiver Operating Characteristic (ROC) curves to give a more comprehensive overview of model performance across all seven classes. It demonstrates the classifier's robustness and discriminative power, particularly in multi‐class environments. Additionally, to summarise performance across all classes, average precision scores and micro‐ and macro‐averaged ROC‐AUC have been computed. Figure 12 represents the ROC curves for each class.

**FIGURE 13:**
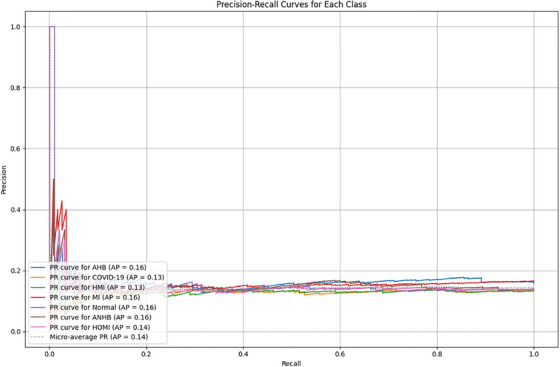
Precision–recall curves for each class.

Figure 13 presents precision recall curves. Every class is treated similarly by the macro‐average. To get the average metric, the micro‐average adds up the contributions from every class. Class imbalance is taken into account by the weighted average. While PR curves show model precision at different recall levels important in class‐imbalanced settings, ROC curves show the trade‐off between true positive and false positive rates. Table 11 provides macro, micro, and weighted averaged metrics.

**TABLE 11:** Standardised Metrics

Metric	Macro	Micro	Weighted
Precision	0.9456	0.9429	0.9459
Recall	0.9429	0.9429	0.9429
F1 score	0.9429	0.9429	0.9431
Accuracy	0.9429	0.9429	0.9429

**FIGURE 14:**
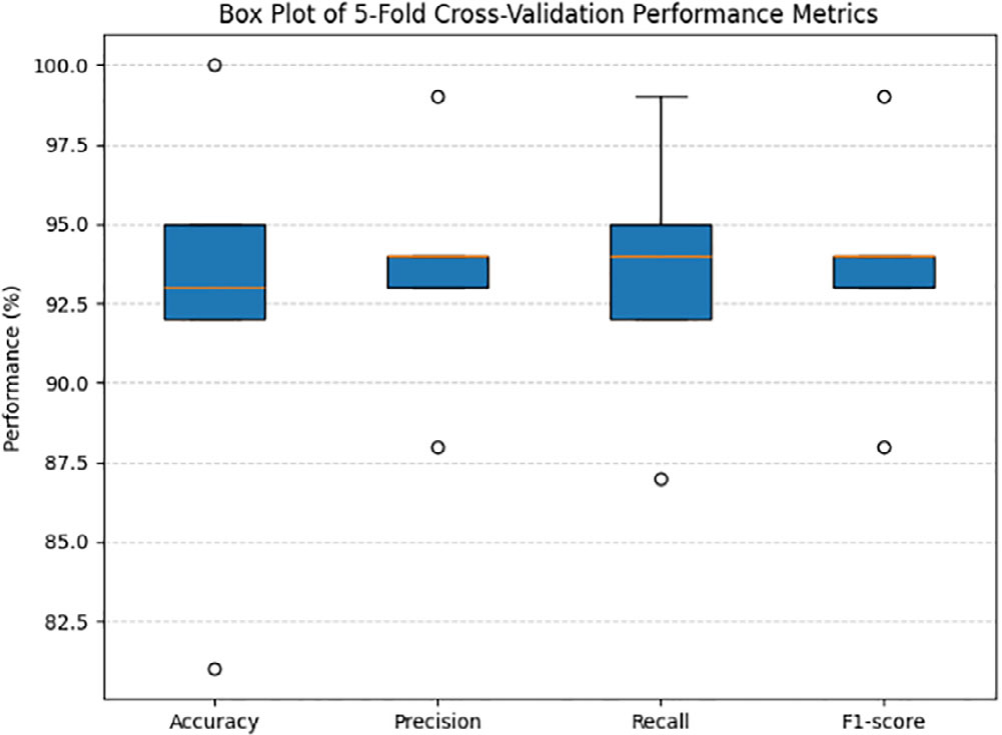
Box plot of 5‐fold cross‐validation performance metrics.

Statistical validation was performed using 5‐fold cross‐validation. The proposed model achieved a mean validation accuracy of 94.34% ± 6.1%, indicating consistent performance across folds. A 95% confidence interval (86.77%–100.00%) was computed to quantify the uncertainty of performance. The relatively wide interval reflects both the high accuracy values as well as the limited number of folds, which is usually common in the medical imaging studies with constrained datasets. Figure 14 represents the box plot which represents inter‐fold variability across evaluation metrics, which demonstrates consistent median performance and acceptable dispersion, which supports the statistical robustness of the proposed model.

A thorough ablation study has been carried out to examine the distinct contributions of important elements incorporated into our suggested multimodal chronic cardiac disease prediction model. An ablation study is a commonly used machine learning technique that involves systematically altering or removing certain architectural or procedural components in order to isolate and assess their impact. Three factors were examined: (i) the modality of the data, (ii) the cross‐validation approach, and (iii) the augmentation of the data. First, a suggested multimodal strategy that combines two different ECG sources has been compared with the impact of using a single ECG dataset. Limited feature representation and class separation were demonstrated by the single‐dataset model, particularly for overlapping cardiac diseases like MI and ANHB. As demonstrated by the validation findings, on the other hand, the multimodal model produced improved class‐specific metrics; for example, MI obtained an F1 score of 0.936, and ANHB improved to 0.949. Better generalisation and discriminative performance across all classes are made possible by the model's capacity to discover complementing patterns from diverse data sources. Second, the function of CV, or K‐Fold cross‐validation has been evaluated. Performance fluctuated between runs and was biased by random train‐validation splits in the absence of CV. The model demonstrated more stable and consistent validation scores across folds when using 5‐fold CV, which helped to ensure robustness and decrease overfitting. Conditions such as HOMI and HMI, for instance, continuously maintained good precision and recall (≥0.94) over several folds, demonstrating how well cross‐validation stabilises model learning and performance estimates. Finally, the impact of data augmentation has been examined, which was added sparingly to the ECG spectrograms in order to replicate the unpredictability of signals in the actual world. Although it was not a significant factor, augmentation prevented the model from overfitting on the comparatively small and structured ECG datasets, which improved generalisation, especially in validation accuracy and F1‐score. Overall class‐wise recall and specificity showed slight but discernible gains, particularly for under‐represented classes. This ablation study demonstrated that multimodal input, K‐fold cross‐validation, and data augmentation all significantly improved the performance of the model during training and validation. Therefore, it makes sense to integrate them in order to guarantee the robustness and dependability of the suggested system for predicting chronic heart disease in the actual world.

In order to improve the prediction of chronic cardiac disease, a number of optimisation strategies have been combined, such as normalisation, data augmentation, K‐fold cross‐validation, and multimodal fusion of dual ECG sources. However, the performance gains that have been seen, including overall accuracy, point to the integrated design's overall efficacy.

Despite its excellent performance, the suggested model may have biases and ethical issues because it uses two datasets from Mendeley and Kaggle. Analysis of age, gender, and ethnicity distributions is hampered by the lack of available metadata, and these datasets could not accurately reflect the general population. When applied to under‐represented subgroups or unobserved clinical contexts, this could lead to differences in performance. Potential misdiagnosis situations raise ethical questions since they may have serious repercussions for patient treatment. More demographically balanced datasets, fairness audits, and the use of clinical specialists to validate model predictions should all be part of future research.

Table 12 provides a comparison of the accuracy of different deep machine learning methods with proposed deep machine learning‐based IoMT‐enabled multimodal. It is clearly shown that the proposed model achieved the highest accuracy of 97.18% as compared to previous state‐of‐the‐art published approaches. A comparison of current deep learning and machine learning methods for predicting chronic heart disease is shown in Table 12. These algorithms have an accuracy range of 75.6% to 96.28%. The data modality, feature representation, and algorithmic complexity all have a contributing role in it. Conventional models, like SVM‐based approaches (85%), banded kernel ensemble techniques (81%), and standalone machine learning algorithms (75.6%) exhibited relatively low performance. This is mainly because they mostly rely on manually created features and are unable to fully capture the intricate temporal and spatial patterns found in ECG signals. Previous CNN and ANN approaches performed moderately (77.3%–91.1%), outperforming classical methods but being constrained by shallow architectures and single modality inputs. Even though they were mostly unimodal and lacked robustness, more complex ensemble strategies, like stacking‐based DNN models (92.23%), deep cardiac learning algorithms (93.96%), and combined random forest with gradient boosted tree methods (96.28%), achieved higher performance through model diversity and enhanced feature learning. On the other hand, because of the successful fusion of complementary ECG modalities that enhanced feature representation and reduced information loss, the suggested multimodal chronic cardiac disease prediction framework achieved an average training accuracy of 97.18%, exceeding prior baselines. Despite these encouraging outcomes, there are a few things to be aware of. First, the study is limited by the size of the dataset and the lack of external multicentre validation, which may impair generalisability across various populations and acquisition circumstances, even though fivefold cross‐validation was used to increase statistical reliability. Second, future research should focus on clinically relevant parameters like sensitivity, specificity, and AUC to better reflect diagnostic value, even when accuracy‐based comparisons show definite performance gains. Furthermore, robustness in the face of real‐world noise and signal artifacts is also a crucial topic for additional research.

**TABLE 12:** Comparison of accuracy with other deep machine learning approaches.

Ref.	Method	Accuracy
[36]	DNN	—
[37]	Deep neural network	83.3
[38]	Random forest, support vector machine, logistic regression, and K‐nearest neighbour	91.80
[39]	Random forest, support vector machine, logistic regression and K nearest neighbour classifier	86.48%
[40]	Hybrid random forest	88.7%
[41]	k‐NN, decision tree, naive Bayes, logistic regression (LR), support vector machine (SVM), neural network, and vote	87.4%
[42]	ANN, SVM, LR, KNN, classification tree and NB	85%
[43]	Hybrid model fuzzy clustering	88.3%
[44]	LR, KNN, ANN, SVM, DT, NB, and RF	89%
[45]	ANN	88%
[46]	Random forest, support vector machine (SVM), and logistic regression	—
[47]	Support vector machine, logistic regression, K‐nearest neighbour, naive Bayes, and ensemble voting classifier	85.49%
[48]	Support vector machine	85.7%
[49]	Naive Bayes, support vector machine (SVM), decision tree, and K‐nearest neighbour (KNN)	88%
[50]	Deep belief network (DBN) enhanced grey‐wolf optimisation‐based feature selection algorithm (EGWO‐FSA).	90%
[51]	deep cardiac learning algorithm	93.96%
[52]	ML algorithm	75.6%
[53]	SVM and Sequential SVM	85%
[54]	PAT algorithm	—
[55]	ANN	89.9%
[56]	CNN	77.3%
[57]	semi‐supervised model (ECGMatch)	—
[58]	banded kernel ensemble technique	81%
[59]	Random forest (RF), K nearest neighbours (KNN), gradient boost, AdaBoost, and XGBoost	93.0%
[60]	Myocardial ischaemic injury index	—
[61]	Machine learning for hypertrophic cardiomyopathy prediction	—
[62]	CNN	91.1%
[63]	Stacking ensemble‐based binary classifier, DNN	92.23%
[64]	Deep learning and classic machine learning	92.9%
[65]	Random forest and gradient Boosted tree	96.28%
[66]	CNN	88.61%
[67]	Deep learning‐based model, CHDdECG	—
**Proposed model**	**Multimodal for chronic cardiac disease prediction**	**97.18%**

Using multimodal datasets, the proposed model offers high‐performance categorisation of several cardiac‐related diseases; however, its clinical utility goes beyond accuracy measures. Contextualising how these outputs support clinical decision‐making is essential for practical application. The system can serve as a helpful tool by highlighting potentially important results, giving anomalous scan review priority, and minimising human supervision errors, all without replacing professionals. This method enables physicians to make quicker and better‐informed judgements, especially in situations with limited resources or high patient loads. More scalable and equitable healthcare delivery may result from the integration of such intelligent systems into clinical decision procedures, which can also improve diagnostic efficiency and consistency. The proposed strategy is made to be easily integrated into current clinical workflows in order to guarantee practical impact. Depending on the healthcare setting, it can be used as a second‐opinion system, which provides cross‐verification for diagnostic consistency, especially in ambiguous or borderline cases; a real‐time alert mechanism, which notifies medical personnel of critical abnormalities during image acquisition or upload; or a triage support tool, which prioritises urgent cases based on automated initial analysis. These positions enhance evidence‐based and time‐sensitive care by enhancing clinician capability rather than interfering with existing procedures. Furthermore, the system's modular design facilitates quick and localised analysis by enabling deployment at edge devices or interaction with hospital PACS/RIS systems. As the suggested model is provided as a conceptual IoMT‐enabled clinical decision support framework which is assessed using publicly accessible ECG datasets from online data repositories, this study did not assess inference latency, energy usage, or hardware‐level deployment empirically. As a result, it represents possible deployment viability rather than verified on‐device performance. In edge‐assisted healthcare settings, where ECG data can be analysed locally and incorporated into current clinical processes to support clinician decision‐making, the feature‐level fusion of the model approach and compact CNN architecture are appropriate. Quantitative assessment of edge devices with an emphasis on inference time, energy efficiency, and end‐to‐end system integration will be part of our future work.

The IoMT healthcare system transforms commonplace physical things and medical gadgets into an intelligent, well‐established healthcare system [85], as the Internet of Medical Things (IoMT) makes it possible to continuously monitor and predict health by visualising a network of medical devices [86]. IoMT and deep machine learning help to improve personalised healthcare, especially for the treatment of long‐term conditions or chronic diseases [87]. Rapid developments in the healthcare industry have been made with the goal of improving patient care and health services [88]. Early detection, high accuracy, and individualised treatment are just a few of the key benefits of the proposed IoMT‐enabled system that utilises deep machine learning to predict chronic diseases, significantly aiding healthcare professionals. These features can enhance patient outcomes and promote preventative care. Additionally, the system reduces the need for repeated hospital visits, thereby lowering healthcare costs and increasing accessibility. Moreover, by streamlining patient care, this approach encourages better resource management in healthcare facilities. While high initial setup costs, concerns about data privacy, and sensor reliability are minor drawbacks, they can be mitigated through careful infrastructure planning.

## 7 Conclusion

A multimodal, IoMT‐enabled intelligent system for healthcare 5.0 is being developed with the goal of correctly and swiftly predicting chronic heart disease. An IoMT‐based method and a multimodal approach are employed to increase the accuracy rate and reaction time. This study's algorithm effectively utilises multimodal ECG datasets to distinguish between pathological and normal cardiac states with a high degree of accuracy. Both databases provide unique diagnostic data but reflect different modalities due to variations in sources, patient demographics, data collection methods, and clinical circumstances. The data was preprocessed to ensure uniformity in format and scale through resizing and normalisation. Images from both datasets were standardised by resizing and normalising pixel values to facilitate effective training. The model architecture included two distinct convolutional neural networks (CNNs) designed to process each dataset separately. The outputs of these CNNs were then concatenated for multimodal integration. K‐fold cross‐validation was employed to ensure robust model performance, addressing variability and potential imbalances in the data. This technique guarantees that each instance in the training set is used once as a validation set while the remaining instances are utilised for training.

Strong model learning occurred during the training phase, as evidenced by the training accuracy of 97.18%, and the validation accuracy was 94.34%, which indicates strong generalisation to untested data. These findings show that the model does a good job of capturing the subtleties of various cardiac diseases, which advances the field of predictive healthcare. A varied representation of ECG signals is ensured by using datasets with numerous subfolders containing distinct cardiac diseases, which enhances the model's ability to differentiate between different cardiac conditions. The performance of this model suggests that it could be valuable in clinical settings for the early identification of cardiovascular disorders. Furthermore, by enabling real‐time monitoring and analysis of heart health, incorporating ideas from Healthcare 5.0 and the IoMT could enhance the usefulness of the model even further. In summary, this work lays the groundwork for future research and advancement in the field of predictive analytics in cardiology and represents a major advancement in the discipline.

In the future, adding more physiological inputs and patient metadata will broaden the model's detection range of cardiovascular disorders. We recognise the value of external validation even though the current study shows encouraging outcomes using two multimodal datasets. In order to further evaluate the model's generalisability and robustness over a range of demographics, acquisition settings, and device standards, future work will concentrate on incorporating a third independent dataset, maybe from a different institution. It is recognised that model interpretability is crucial for clinical usage. Explainability techniques like Grad‐CAM, SHAP, and LIME to display contributing ECG areas and promote clinical trust will be explored in future research, and the model practicality through edge deployment scenarios or simulations, taking into account factors like inference time, model size, and resource needs, will be evaluated. Furthermore, improving the interpretability of the model using sophisticated methods such as attention processes or feature importance analysis will make it easier for medical professionals to embrace it. In the future, to improve patient‐specific predictions and treatment plans, the main focus will be on guaranteeing generalisability across various datasets and clinical settings, combining continuous learning techniques, integrating uncertainty estimation methods, improving prediction reliability, and leveraging EHR for personalised medicine.

## Author Contributions

R. J. and T. A. collected data from different resources. R. J., T. A., A. S., A. A. S., and K. M. A. performed the formal analysis and simulations. R. J., T. A., and S. A. wrote the original draft. S. A., A. A. S., and K. M. A. reviewed and edited the manuscript. T. A. and K. M. A. supervised the study. R. J. and A. S. prepared the figures and tables. T. A., S. A., and K. M. A. revised the manuscript and improved the overall quality. All the authors have read and agreed to the published version of the manuscript.

## Funding

The authors have nothing to report.

## Conflicts of Interest

The authors declare no conflicts of interest.

## Data Availability

To encourage repeatability and transparency, the source code and related scripts used in this study are made available on GitHub. In accordance with the procedures described in this article, for additional research, view the code at https://github.com/786RabiaKhan/Chronic‐Cardiac‐Disease‐Prediction‐Multimodal.git.
